# Managing Contextual Complexity in an Experiential Learning Course: A Dynamic Systems Approach through the Identification of Turning Points in Students' Emotional Trajectories

**DOI:** 10.3389/fpsyg.2017.00667

**Published:** 2017-05-03

**Authors:** Gloria Nogueiras, E. Saskia Kunnen, Alejandro Iborra

**Affiliations:** ^1^Department of Educational Sciences, University of AlcaláAlcalá de Henares, Spain; ^2^Department of Developmental Psychology, University of GroningenGroningen, Netherlands

**Keywords:** contextual complexity, cognitive conflict, complexity management, emotional trajectories, dynamic systems, turning points, Monte Carlo permutation tests, process-oriented experiential learning

## Abstract

This study adopts a dynamic systems approach to investigate how individuals successfully manage contextual complexity. To that end, we tracked individuals' emotional trajectories during a challenging training course, seeking qualitative changes–turning points—and we tested their relationship with the perceived complexity of the training. The research context was a 5-day higher education course based on process-oriented experiential learning, and the sample consisted of 17 students. The students used a five-point Likert scale to rate the intensity of 16 emotions and the complexity of the training on 8 measurement points. Monte Carlo permutation tests enabled to identify 30 turning points in the 272 emotional trajectories analyzed (17 students ^*^ 16 emotions each). 83% of the turning points indicated a change of pattern in the emotional trajectories that consisted of: (a) increasingly intense positive emotions or (b) decreasingly intense negative emotions. These turning points also coincided with particularly complex periods in the training as perceived by the participants (*p* = 0.003, and *p* = 0.001 respectively). The relationship between positively-trended turning points in the students' emotional trajectories and the complexity of the training may be interpreted as evidence of a successful management of the cognitive conflict arising from the clash between the students' prior ways of meaning-making and the challenging demands of the training. One of the strengths of this study is that it provides a relatively simple procedure for identifying turning points in developmental trajectories, which can be applied to various longitudinal experiences that are very common in educational and developmental contexts. Additionally, the findings contribute to sustaining that the assumption that complex contextual demands lead unfailingly to individuals' learning is incomplete. Instead, it is how individuals manage complexity which may or may not lead to learning. Finally, this study can also be considered a first step in research on the developmental potential of process-oriented experiential learning training.

## Introduction

This study is based on two assumptions: first, that learning and development occur in response to contextual demands that challenge individuals' ways of meaning-making and lead to the creation of more adapted ones (Piaget, 1975/1985); second, that individuals' encounters with conflicting contextual demands are usually associated with the experience of negative emotions (Frijda, 1986; Carver and Scheier, 1990; Inzlicht et al., 2015). Within this framework, we find that a promising way to grasp individuals' successful management of challenging environmental demands is to track their emotional experience over time from a Dynamic Systems perspective (Thelen, 1989; Van Geert, 1994; Kunnen, 2012). This process-oriented approach supports the idea that it is the dynamic patterns of positive and negative emotions over time which can be positive or negative for learning (Sansone and Thoman, 2005). Our assertion is that individuals' initial emotional responses to conflicting and potentially unpleasant demands will be replaced by a qualitatively different and more pleasant emotional response, in the event of successful management. This change will be indicated by turning points (Hayes et al., 2007; Eubanks-Carter et al., 2012) in the individuals' emotional trajectories. We also assume that a particularly complex contextual input may be a trigger for the successful management of complexity.

As higher education teachers, we find process-oriented experiential learning methodologies to be particularly appropriate for challenging individuals' ways of meaning-making, and thus for contributing to their learning and development. In this study we therefore focused on the 17 participants in a 5-day higher education course based on process-oriented experiential learning. We aimed to investigate how these students successfully managed contextual complexity by tracking their emotional trajectories during the training, in search of turning points and determining their relationship with perceived training complexity.

### Cognitive conflict as a trigger for meaning-making

From a constructivist standpoint, human beings make meaning of reality by creating our own personal theories or models of the world, which give us a provisional framework for understanding. As a result, meaning-making entails a dynamic process of continuous updating of these models in order to create adapted responses to an ever-changing environment (Piaget, 1975/1985). This process is triggered by conflicts that arise in the event of discrepancies between our experience and our model of the world, or in other words, between our way of creating meaning and the results we obtain (see Piaget, 1952, and cognitive disequilibrium).

Conflicts are acknowledged as being the trigger for learning (Piaget, 1975/1985), although whether this happens depends on the strategies we adopt to cope with those conflicts (Kunnen, 2006). In Piagetian terms, when we are first confronted with a conflict, our most economical reaction is to try to solve it through assimilation. In other words, we change our interpretation of the situation, or if possible, the situation itself, so that it once again fits in with our model of the world. The more challenging response of accommodation is only applied if assimilation is unsuccessful. Accommodation entails making significant changes in our model of the world, which reduces the discrepancy between our way of meaning-making and the contextual demands. These accommodational changes lead to learning and development (Kunnen and Bosma, 2000). By contrast, if we are unable to resolve the discrepancy, our confidence in our way of creating meaning is undermined and the potential for learning is narrowed.

### Cognitive conflict and negative emotions in learning contexts

Emotions enhance our meaning-making processes by boosting what we attend to and by providing us guidance for adaptive action (Frijda, 1988; Lazarus, 1991; Solomon, 2007; Bradley, 2009). In particular, conflictive situations tend to be linked with negative emotions (Frijda, 1986; Carver and Scheier, 1990; Inzlicht et al., 2015). In this context, Kunnen and Wassink (2003) state that unpleasant emotions are drivers for learning, as they motivate us to react in order to reduce the discrepancy between the meaning we create in a given situation and the demands of that situation. However, like conflict, emotional arousal does not automatically lead to learning (Weiss, 2000). Instead, emotions may either impede or motivate learning depending on how individuals become aware of those emotions and how they manage them (Taylor and Cranton, 2013). As an example, D'Mello and Graesser (2011) argue that “it is not confusion itself, but the effortful cognitive activities aimed at resolving the confusion that presumably are beneficial to learning” (p. 1307).

Research in higher education has proved that learning settings that lead students to question their accepted ways of knowing tend to be unsettling (Kegan, 1994; Antonacopoulou and Gabriel, 2001; Cranton, 2002; Apte, 2009; McEwen et al., 2010) and elicit emotions such as fear, grief, and anger (Dirkx et al., 2006; Dirkx, 2011). As examples, the following studies investigated the relationship between conflicting learning demands and students' unpleasant emotional experiences: D'Mello et al. (2009) found a predominance of confusion and frustration in a learning context that was aimed at facilitating deep learning; Nogueiras et al. (2016), and Nogueiras and Iborra (2016) found disorientation, insecurity and frustration as a common initial response to a training course that promoted students' self-direction.

### Studying emotions from a dynamic systems perspective: identifying turning points

It has long been widely accepted that negative emotions are bad for learning and positive emotions are good for learning, to the extent that the former should be controlled or eliminated (Lepper and Henderlong, 2000; Noddings, 2003). However, some authors (see for example D'Mello et al., 2014) argue that this assumption is simplistic and inaccurate. Interestingly, Sansone and Thoman (2005) point out that “it is the dynamic patterns of positive and negative emotions at certain points in time in a given context what can be considered good or bad for learning” (p. 509). This emphasis on dynamic patterns is at odds with the widespread static approach to the study of emotions, which is based on cross-sectional research designs underlined by a unidirectional model of causation. However, if emotions are considered to be processes that dynamically evolve over time due to interactions between individuals and context (Fogel et al., 1992; Barrett, 2009; Frijda, 2009), a paradigm shift is required. This paradigm shift points toward process-oriented approaches that enable the study of within-person emotional patterns of change (Larsen et al., 2009; Scherer, 2009; Kuppens et al., 2010). Dynamic systems theory is particularly suited to this approach (Camras and Witherington, 2005; Lewis, 2005; Lichtwarck-Aschoff et al., 2009).

Dynamic systems theory is a metatheoretical framework for understanding developmental processes, which are conceived as non-linear dynamic systems (Witherington, 2007; Van Geert and Van Dijk, 2015). These systems are formed by interconnected and interacting components that affect each other and develop over time, due to interactions between individuals and their context (Thelen, 1989; Van Geert, 1994; Kunnen, 2012). As developmental processes are characterized by sudden changes and irregularities, their study requires methodologies that enable to grasp variability and change while they occur, rather than comparing pre- and post-change behavioral patterns (Van Geert and Van Dijk, 2002; Van Dijk and van Geert, 2007; Fogel, 2011). A dynamic systems approach therefore uses individual microdevelopmental trajectories^1^ as the unit of analysis, and examines them using as many measurements over time as possible (Molenaar, 2004; Siegler, 2006; Yan and Fischer, 2007).

Qualitative changes in individual microdevelopmental trajectories can be identified based on the concept of the turning point. Turning points are points that mark meaningful deviations in trends in time series data that involve discontinuous changes (Hayes et al., 2007; Eubanks-Carter et al., 2012). These changes entail a transition from one variability pattern to another variability pattern (Kunnen et al., 2012) in such a way that the trajectories separated by a turning point differ in direction or nature (Abbott, 1997).

### Meaning-making from a dynamic systems perspective

From a dynamic systems perspective, contexts contribute to the emergence of individuals' behavior by providing both constraints and opportunities (Thelen and Ulrich, 1991; Van Geert, 1994; Vallacher et al., 2015). The greater the contextual variability, the greater the likelihood of experiencing conflicts that lead us to make changes to adapt (Bosma and Kunnen, 2001; Hayes et al., 2015). In other words, contextual variability offers systems room to explore and to adapt to new situations (Thelen and Smith, 1994; Van Geert, 1994). As a result, if learning and development is to occur, a factor must challenge our patterns of meaning-making so that they are reorganized on a more complex level (Thelen, 2005; Kloep et al., 2009). Within this framework, and in line with Piaget's argument, the stability of our model of the world due to the maintenance of our meaning-making patterns would entail assimilation, whereas the destabilization of our model of the world and the emergence of new meaning-making patterns would entail accommodation. In this sense, the occurrence of any variability in developmental trajectories of any kind—such as emotional trajectories—enables us to identify developmental transitions (Van der Maas and Molenaar, 1992; Granott et al., 2002).

### Experiential learning as a source of cognitive conflict

Our understanding of experiential learning differs from the model proposed by Kolb (1984) and Kolb and Kolb (2009). This model is based on a learning cycle that starts with students' reflection on the content of concrete experiences in order to create abstract concepts, which are then tested by active experimentation. This in turn generates further concrete experiences. By contrast, and based on the work of McWhirter (2002), we argue that the key to experiential learning is to use of students' own sensory and natural experiences to subsequently structure them using detailed modeling distinctions. As a result, in the “process-oriented experiential learning model”^2^ (McWhirter, 2002), the emphasis is not on the creation of abstract ideas or explanations of personal experiences–based on the content of the experience, or *what* happens. On the contrary, the emphasis is on the creation and exploration of formal distinctions that are then tested as they may be useful in making sense of the individual's experiences in a more complex way—considering the whole experience from a process perspective, or *what, how* and *why* it happens.

In specific terms, the experiential learning model that we present is based on the following sequence: (1) Creating an experience: students undertake an open exploration of their natural experience or intuitive understanding about the phenomena being studied; (2) Reviewing: students share the range of experiences they obtained in the exploration, identifying similarities and differences in comparison with other classmates' experiences—or even with other previous personal experiences; (3) Formalizing: the trainer introduces formal models and distinctions; (4) Testing: students compare and test the formal model and distinctions against their own personal and group experience.

The experiential learning model differs from the traditional learning model, labeled didactic learning, which is based on the following sequence: (1) Description of a formal model: the trainer tells; (2) Demonstration: the trainer shows; (3) Experience: the students do; (4) Provision of feedback: the trainer tells the students what went wrong and right. This sequence is useful for rote learning and learning protocols, so that there is an increase in “knowing” before the uncertainty and risk involved in “doing”. However, it overlooks the fact that ready-made techniques do not correspond with reality, which has variations that are ignored in favor of the illusion of certainty that is provided by protocols. The didactic learning model therefore leads to a reduction toward the “correct” way, and fosters a dependent, repetitive and unquestioning style of learning that prevents students from engaging in exploration, creativity, and self-direction.

By contrast, experiential learning as understood by McWhirter (2002) contributes to developing the competence of learning to learn, increases the depth of learning, encourages an attitude of curiosity and wonder, and prepares students to take what they learn into the world. Indeed, this learning sequence is similar to the one we all naturally follow as children when making meaning of our surroundings: starting from a baseline of not knowing, we build an understanding. However, the application of an experiential learning sequence also entails little security in “knowing,” because there is no “right answer” or example to begin with. This might initially lead students to feel insecure and uncomfortable, as they have to develop an open orientation to new experiences. Furthermore, if students continue to apply the didactic learning sequence they are accustomed to, there may have an additional sense of not “knowing,” while believing that they should know before they continue to learn. Apart from the learning sequence itself, the contents explored in process-oriented experiential exercises also tend to be complex and challenging for students.

A formal model that can be useful for illustrating students' typical responses to experiential learning when they are first exposed to it is the Three *sets model: set-up-upset-set-down* (McWhirter, 2000). This model describes three stages in the process of meaning-making of a conflictive experience. The students' initial set-up would be the learning sequence that they are used to, and which they expect to find at the beginning of the training—the didactic sequence in most cases. The upset would be the destabilization experienced by the students caused by the challenging demands of the experiential sequence and the exploration of personal experiential content. The set-down would involve potential changes made by the students in response to the upset. This set-down could potentially be related to cognitive accommodation.

In view of the above, the hypothetical emotional trajectory of students in a process-oriented experiential learning context may be as follows. First, the students' upset at the conflicting training demands could lead them to the experience of intense negative emotions and a low level of positive emotions. Later, the students' development of new meaning-making strategies for coping with the challenging demands would entail a decline in the intensity of negative emotions and an increase in the intensity of positive emotions. This could be identified through the occurrence of turning points in emotional trajectories.

It is important to note the positive aspect of the upset in a process-oriented learning setting, and how students are also supported to learn to learn in this way. In fact, the experiential methodology itself revolves around learning management, which in this methodology is considered as something that can be taught and learnt. In fact, this learning model is intended to facilitate qualitative changes in students' way of learning, and specifically in the way they organize information while they learn. In this process, both the recognition and the management of the negative emotions that are expected to be experienced as a response to contextual challenge are assumed to enhance individuals' ability to manage learning effectively.

### The present study

The main goal of the present study is to investigate the process of successfully managing contextual complexity by the 17 participants in a 5-day higher education course, based on a process-oriented experiential learning model. To meet this goal, we aimed to: (1) identify turning points in the participants' positive and negative emotional trajectories, (2) test whether those turning points coincide with periods in the training perceived by participants as particularly complex. We also aimed to test the widespread assumption that contextual complexity leads to the destabilization of individuals' ways of meaning-making, and consequently to learning.

## Methods

### The learning context studied

#### Description of the training course

This study focuses on an intensive training course based on a process-oriented experiential learning model (McWhirter, 2002). The course took place in a Faculty of Education at a Spanish university. It was part of a summer training program for both university students and non-students. The training course lasted 31.5 h over five consecutive sessions. The first four sessions lasted for 7 h, and the fifth lasted for 3.5 h. The training was conducted by the developer of Developmental Behavioral Modeling (DBM)^3^ McWhirter (2011).

The course was entitled *Self-created learning throughout life*, and aimed to develop participants' life competences of self-managing and self-directing their own learning processes. Participants were therefore expected to: (1) gain a deeper understanding of the learning to learn process by modeling, developing, and assessing their autonomous learning processes; (2) explore changes in beliefs, values, self-concept, identity or vital aspirations that take place as a consequence of self-created learning. The reason why we selected this training course for this study was that both the course content (how to manage learning) and the experiential learning methodology (intended to support students' in their improved management of learning) were expected to be highly upsetting and challenging for the participants. This made the training ideal for examining adaptation to cognitive conflict.

The first session of the course started with the participants' exploration of their current understanding about learning. The next sessions started with a review by the participants of the content covered and the exercises performed during the previous session, in order to reconnect with the experience, share their understandings, make connections and identify issues for clarification. The remainder of all the sessions consisted of: (1) the trainer's proposal of experiential exercises and the students' performance of them, with an emphasis on trying different ways of doing things and on extending one's own attention; (2) sharing experiences in order to identify what was similar and what was different, fostering the students' awareness of variety, (3) the trainer's feedback, (4) the trainer's introduction of formal models and process distinctions so that the students could explore their experience with further direction.

During the experiential exercises, three processes were encouraged: (a) to *attend* (playing with attention and moving it); (b) to *notice* (paying attention to the content of what one notices and to the way in which one notices: active or passive, detailed or not, rigid or open); (c) to *explore* and to *investigate* (going beyond what is initially recognized, opening things up and creating things that one does not know). By way of an example, an experiential sequence which was used on the course to explore different personal learning experiences consisted of the following steps: (1) Identifying the experience of learning to be explored; (2) Identifying the resources available before the experience, both from the environment and from oneself; (3) Reviewing what happened throughout the learning sequence, paying attention to what was changing and what one was doing; (4) Reviewing how one checked that the learning had happened; (5) Thinking about further development, in terms of changes that could be introduced to improve learning.

#### Participants

The participants on course were 31 higher education students and professionals, mostly from educational, psychological, and health disciplines. At the beginning of the course, the participants were informed about the research and the confidentiality thereof, and all contributed information and agreed to take part by means of informed consent.

The sample in this study consisted of 17 participants (12 women, mean age 33.53, age range 19–55) who completed all the measurements during the course. All the participants lived in Spain, except one who lived in United Kingdom and came to Spain for the training. There were 13 participants from Spain, 1 from Ireland, 1 from Mexico, 1 from the United Kingdom and 1 from Romania. Of the 17 participants, 7 were undergraduate students on either Teacher Training or Psychology courses, 2 were doctoral students in Education, 2 were psychologists, 2 were Psychology university lecturers, 1 was a Secondary Education English teacher, 1 was a librarian, 1 was a veterinarian and 1 was a doctor.

### Data collection

The first author of this article attended the course as a participant, while the third author was an active observer of the training. The two authors and the trainer were responsible for the data collection. Within the framework of a more extensive data collection, the focus in this article is on a follow-up questionnaire distributed over eight measurement points (m.p.) during the training course, i.e., twice per session in the first four sessions. The follow-up questionnaires were strategically distributed at the beginning and at the end of the session or at particular points that were expected to be more challenging for the course participants, so that the potential emotional arousal associated with them was more likely to be recorded. See Appendix 1 in Supplementary Material for an overview of the timeframe of the training course, including the distribution of questionnaires.

In each questionnaire, the participants self-reported the intensity of their emotions and the degree of perceived training complexity that they experienced at that point on the course.

The intensity of the emotions was assessed by means of a list rated on a Likert scale, from very low (1) to very strong (5). The list was designed by the first and the third authors of this article with the trainer, and included a range of emotions that they had observed in previous process-oriented experiential learning training activities they had led. The 16 emotions in the list were: joy, sadness, anger, fear, enjoyment, interest, distress, boredom, hope, overwhelmed, overload, confusion, enthusiasm, dissonance, ignorance, and curiosity.

The degree of perceived training complexity was assessed using two items, rated on a Likert scale from very low (1) to very strong (5). The items were: (a) conceptual complexity, which referred to the complexity associated with the students' understanding of the formal models introduced by the trainer, (b) performative complexity, which referred to the complexity involved in doing the experiential exercises, in terms of both their structure and the personal experiential content explored by students.

### Data analysis

In order to systematize the analysis of the emotions assessed by the participants, each emotion was coded as either positive or negative, which resulted in: (a) 6 positive emotions (joy, enjoyment, interest, hope, enthusiasm, curiosity), and (b) 10 negative emotions (sadness, anger, fear, overwhelmed, boredom, distress, overload, confusion, dissonance, ignorance). Emotional trajectories were created for each participant and for each emotion, which consisted of the series of intensity scores provided for the eight measurement points. This means that the trajectories are nested, i.e., there are several emotional trajectories for each individual.

For the analysis of the complexity scores, an overall complexity score for every participant was computed for each measurement point by averaging the scores given in the two items included in the questionnaires—conceptual complexity and performative complexity. This provided a score for complexity for each student and for each measurement point, with a range of between 1 and 5.

#### Identifying turning points

Looking for an unexpectedly large peak in the data has proved to be effective in identifying discontinuous patterns showing the emergence of qualitative changes in individual developmental trajectories, (see for example Van Geert and Van Dijk, 2002; Van Dijk and van Geert, 2007, 2011). This technique inspired us when we designed our procedure to identify turning points in the participants' emotional trajectories. This procedure consisted of two steps. First, the trajectories for the 16 emotions scored by the 17 participants (i.e., 272 emotional trajectories: 102 positive and 170 negative) were examined for points that fell outside a computed confidence interval. These were labeled as exceptional points. Second, exceptional points were examined using Monte Carlo permutation tests to determine whether they showed a qualitative change in the pattern of the emotional trajectory. In this case they were labeled as turning points. The complete procedure is detailed below.

##### The first step: looking for exceptional points

The regression line underlying every emotional trajectory was first computed. Based on the values of that regression line, a confidence interval of 1.65 standard deviations around the spread of the data was computed, so that the upper control limit (UCL) of the confidence interval was 1.65 standard deviations above the regression line, and the lower control limit (LCL) of the confidence interval was 1.65 standard deviations below the regression line.^4^ The points on the emotional trajectories which fell outside the computed confidence intervals were labeled exceptional points.

**Figure 1:**
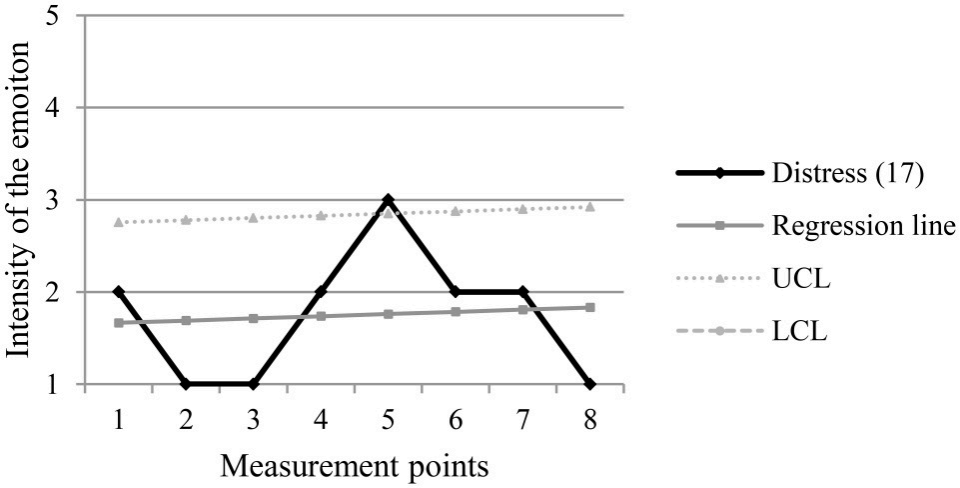
**Example of an exceptional point (m.p.5)**. UCL, Upper Control Limit; LCL, Lower Control Limit.

Table 1 shows an example of the computing of the confidence interval of the scores for intensity of distress of one of the participants throughout the eight measurement points (m.p.). In the example, m.p. 5 is an exceptional point, which means that the score in the intensity of distress in that point is greater than the value of the upper control limit of the computed confidence interval. Figure 1 graphically presents the emotional trajectory for distress, the underlying regression line and the upper and lower control limits of the confidence interval computed from the regression line. This graph shows the exceptional point at m.p. 5, which can be seen above the upper control limit.

**Table 1:** **Example of the computation of the confidence interval for the scores in the intensity of distress of one of the participants**.

**m.p**.	**Scores in distress (17)**	Regression line (intercept + slope) ^*^ data	**Variance (data-regression line)^ˆ^2**	**Confidence interval**	
				**UCL (regression line + 1, 65 σ)**	**LCL (regression line – 1, 65 σ)**
1	2	1.666	0.111	2.754	0.579
2	1	1.690	0.477	2.778	0.603
3	1	1.714	0.510	2.802	0.627
4	2	1.738	0.068	2.826	0.650
5	**3**	1.762	1.533	**2.849**	**0.674**
6	2	1.787	0.046	2.873	0.698
7	2	1.809	0.0363	2.897	0.722
8	1	1.833	0.694	2.921	0.746
		Average variance	0.434		
		Standard deviation (σ)	0.659		

*Data associated to the exceptional point (scores in distress, UCL and LCL), located in m.p. 5, are boldfaced*.

##### The second step: determining which exceptional points are turning points

Once the exceptional points were identified, a statistical analysis was performed in order to determine whether they indicated a qualitative change in the emotional trajectory, i.e., a turning point. The analysis consisted of a comparison of the slope^5^ of the emotional trajectory before and after the exceptional point, in search of significant differences. To that end, Pop tools (Hood, 2010) in Microsoft Excel 2010 were used to perform Monte Carlo permutation tests (see Todman and Dugard, 2001). These tests are also known as random permutations, random sampling techniques or resampling techniques, and are included in the family of bootstrap techniques (Efron and Tibshirani, 1993; Good, 1999). Resampling techniques are well-suited to longitudinal research, and have great explanatory value for small or skewed samples, and result in reliable *P* values, since they do not assume any underlying distribution, or a minimum sample size (for this argument see Van Geert et al., 2012). Standard tests such as *t*-tests are not allowed in these cases (Kunnen, 2006).

Monte Carlo permutation tests estimate the chances that an observed result is caused by chance alone. They compare an empirical distribution of data with a random distribution that is created by reshuffling the empirical data in accordance with a null hypothesis. In this case, the null hypothesis stated that there was no significant difference between the slopes of an emotional trajectory before and after an exceptional point. The reshuffling computes all possible re-orderings of the empirical data set, by computing a very large number of accidental distributions and counts how often the observed or a bigger difference occurs in the random distributions. In this case, the reshuffling counted how often the difference between slopes was the same or bigger than the observed difference. This frequency is then divided by the number of random samples in order to produce a *P* value for the tested difference, which is the probability of the observed difference occurring in the random distributions of the data. If the probability is low, this means that the observed difference is not due to chance and therefore that it is a legitimate difference (for more detail, see Van Geert et al., 2012). In this analysis, 10.000 random distributions were computed and a *P* < 0.05 was considered significant.

#### Testing the relationship between turning points and perceived training complexity

The next step in the analysis consisted of testing whether the perceived complexity of the training was significantly higher at the measurement points where at least one turning point in some emotional trajectories had been identified. Monte Carlo permutation tests were performed to that end. The average complexity score for the measurement points where turning points had been identified and the overall complexity score of the measurement points where no turning points had been identified was computed in order to test whether the former was higher than the latter. The perceived complexity scores of each participant at every measurement point were then reshuffled, and the overall complexity score at the measurement points with turning points, the average score of complexity in measurement points without turning points and the difference between both were then computed. Monte Carlo simulations (10.000 random distributions computed) were used to test whether the difference in the degrees of perceived complexity was significant (a *P* < 0.05) or due to chance.

## Results

### Detection of turning points

#### The first step: identification of exceptional points

In the 272 emotional trajectories analyzed, 142 exceptional points were identified, i.e., 52% of the emotional trajectories had at least one exceptional point. There were 53 exceptional points in positive emotion trajectories (52% of these trajectories), and 89 exceptional points in negative emotion trajectories (52% of these trajectories).

Two types of exceptional points were identified: (1) High exceptional point: a point on an emotional trajectory above the upper control limit of the trajectory's confidence interval; (2) Low exceptional point: a point on an emotional trajectory below the lower control limit of the trajectory's confidence interval.

Low exceptional points were the most common in positive emotion trajectories (62%) and high exceptional points were the most common in negative emotion trajectories (75%). These two types accounted for 70% of the total number of exceptional points (23 and 47% respectively). The amount and percentage of high exceptional points and low exceptional points in positive and negative emotion trajectories are presented in Table 2.

**Table 2:** **Amount and percentage (rounded) of high and low exceptional points**.

**Type of exceptional points**	**Exceptional points in positive emotions trajectories**			**Exceptional points in negative emotions trajectories**		
	**No**.	**Percentage in positive emotions trajectories**	**Percentage in the total no. of trajectories**	**No**.	**Percentage in negative emotions trajectories**	**Percentage in the total no. of trajectories**
High exceptional points	20	38	14	67	75	47
Low exceptional points	33	62	23	22	25	16
Total	53	100		89	100	

#### The second step: detection of turning points

30 of the 142 exceptional points (21%) were identified as turning points. This meant that 11% of the total number of emotional trajectories analyzed (272) had a turning point. Of the 30 turning points, 12 turning points were found in positive emotion trajectories (12% of positive emotion trajectories) and 18 turning points were found in negative emotion trajectories (11% of negative emotion trajectories).

Two types of turning points were identified: (1) Pre-decrease turning point: a point on an emotional trajectory above the upper control limit of the confidence interval of the trajectory, indicating a change in the trajectory from an increasing pattern in the intensity of the emotion toward a decreasing pattern; (2) Pre-increase turning point: a point on an emotional trajectory below the lower control limit of the confidence interval of the trajectory, indicating a change in the trajectory from a decreasing pattern in the intensity of the emotion toward an increasing pattern.

Pre-increase turning points were the most common in positive emotion trajectories (83%), while pre-decrease turning points were the most common in negative emotion trajectories (83%). These two types accounted for 83% of the total number of turning points (33 and 50% respectively). The amount and percentage of pre-decrease turning points and pre-increase turning points in positive and negative emotion trajectories are presented in Table 3.

**Table 3:** **Amount and percentage (rounded) of pre-decrease and pre-increase turning points**.

**Type of turning points**	**Turning points in positive emotions trajectories**			**Turning points in negative emotions trajectories**		
	**No**.	**Percentage in positive emotions trajectories**	**Percentage in the total no. of trajectories**	**No**.	**Percentage in negative emotions trajectories**	**Percentage in the total no. of trajectories**
Pre-decrease turning points	2	17	7	15	83	50
Pre-increase turning points	10	83	33	3	17	10
Total	12	100		18	100	

**Figure 2:**
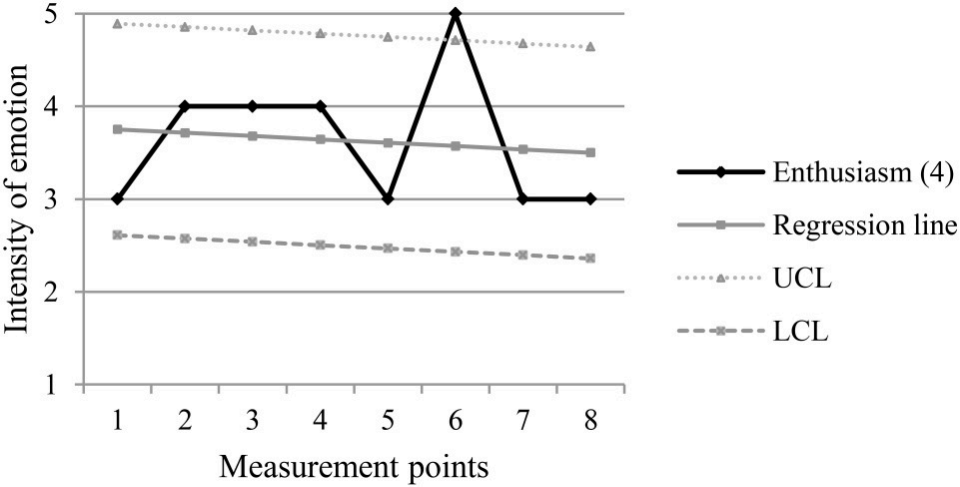
**Example of a pre-decrease turning point (m.p.6) in a positive emotion trajectory**. UCL, Upper Control Limit; LCL, Lower Control Limit.

**Figure 3:**
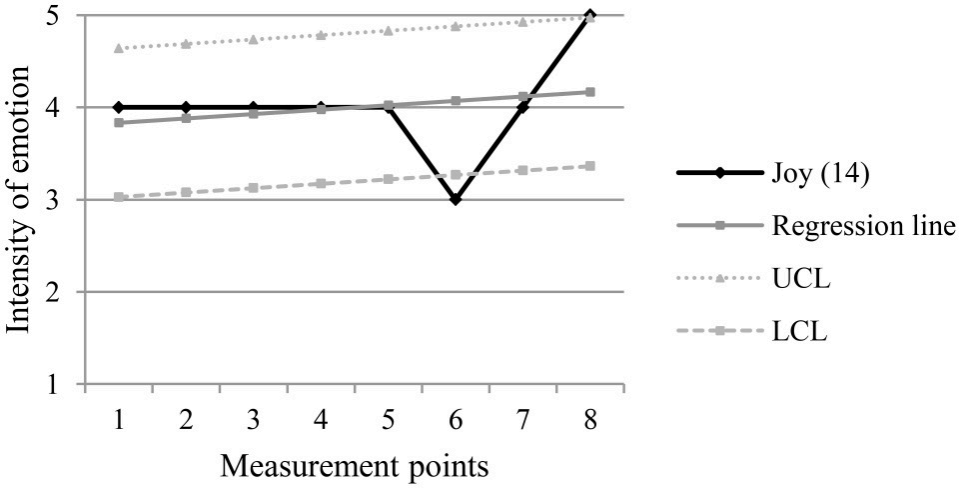
**Example of a pre-increase turning point (m.p. 6) in a positive emotion trajectory**. UCL, Upper Control Limit; LCL, Lower Control Limit.

**Figure 4:**
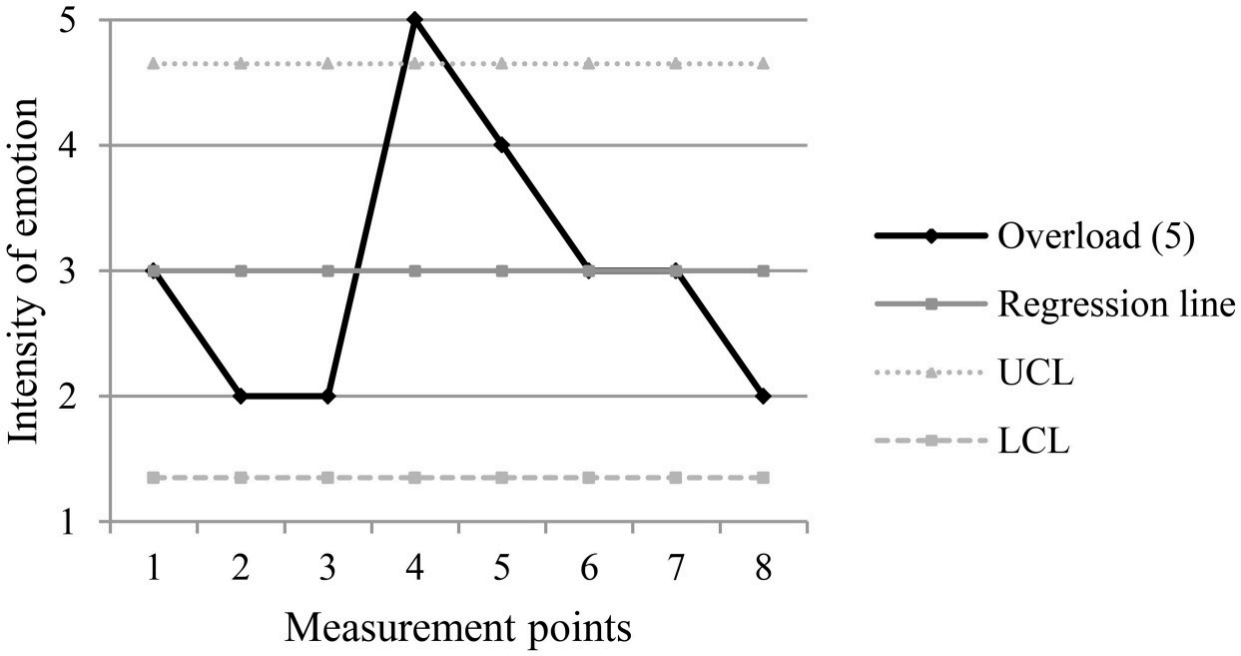
**Example of a pre-decrease turning point (m.p. 4) in a negative emotion trajectory**. UCL, Upper Control Limit; LCL, Lower Control Limit.

**Figure 5:**
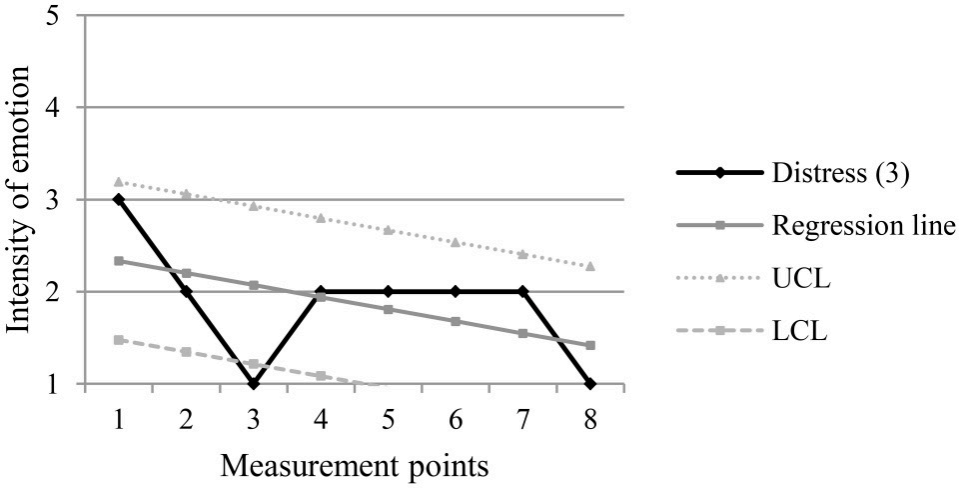
**Example of a pre-increase turning point (m.p. 3) in a negative emotion trajectory**. UCL, Upper Control Limit; LCL, Lower Control Limit.

The following figures (Figures 2–5) are examples of the two types of turning points, pre-decrease and pre-increase, in positive and negative emotion trajectories.

The 30 turning points were distributed over 13 of the 17 participants (76%) and 9 of them presented pre-increase turning points in positive emotions, pre-decrease turning points in negative emotions, or both, which could be considered positively trended turning points. A high percentage of turning points (63.33%) were located at m.p. 4 (9 turning points: 3 pre-decrease turning points in negative emotions, 4 pre-increase turning points in positive emotions, and 2 pre-increase turning points in negative emotions), and at m.p. 6 (10 turning points: 1 pre-decrease turning point in a positive emotion, 3 pre-decrease turning points in negative emotions, and 6 pre-increase turning points in positive emotions). A precise and detailed distribution of the turning points for the participants and for each measurement point is given in Appendix 2 (Supplementary Material).

### The relationship between turning points and perceived training complexity

The measurement points at which turning points were identified had significantly higher complexity scores than the other measurement points (*p* = 0.002). More detailed results were found when the pre-decrease turning points and pre-increase turning points were analyzed separately in both positive and negative emotion trajectories: significantly higher levels of complexity were found at the measurement points with pre-increase turning points in positive emotion trajectories (*p* = 0.003) and at the measurement points with pre-decrease turning points in negative emotion trajectories (*p* = 0.001). Table 4 shows the average and the range of the complexity scores for each measurement point. Table 5 shows detailed results for the differences in complexity scores between the measurement points with turning points and the measurement points without turning points. An overview of the trajectories of the complexity scores for each student over the 8 measurement points is provided in Appendix 3 (Supplementary Material).

**Table 4:** **Average and range of complexity scores for each measurement point**.

	**Measurement points**							
	**1**	**2**	**3**	**4**	**5**	**6**	**7**	**8**
Average complexity scores	2.450	2.980	2.996	3.245	3.176	3.086	3.133	3.279
Range complexity scores	1.5–5	1.5–4	2–5	2–5	2.5–5	1.5–5	2.5–5	2–5

**Table 5:** **Significant differences in complexity scores between m.p. in which turning points (t.p.) were identified and m.p. in which no turning points were identified**.

**Types of turning points**	**Scores in complexity (scale 1 to 5)**				**Difference between mean scores in complexity of m.p. with and without t.p**.	**Scores in complexity higher in m.p. with t.p. than in m.p. without t.p. (*p*)**
	**M.p. with t.p**.		**M.p. without t.p**.			
	**Mean**	**Std dev**	**Mean**	**Std dev**		
All	3.731	0.629	3.162	0.78	0.569	0.002^**^
Pre-decrease t.p. in positive emotions	3.250	0.25	3.231	0.793	0.019	0.562
Pre-increase t.p. in positive emotions	4.250	0.433	3.201	0.776	1.049	0.003^**^
Pre-decrease t.p. in negative emotions	3.889	0.489	3.191	0.783	0.698	0.001^**^
Pre-increase t.p. in negative emotions	3.50	0.25	3.231	0.793	0.269	0.533

** *p < 0.01*.

### Summary of results

11% of the emotional trajectories analyzed had a turning point. The two most common types were: (1) pre-increase turning points in positive emotion trajectories (83% of the turning points in these trajectories); (2) pre-decrease turning points in negative emotion trajectories (83% of the turning points in these trajectories). These two types of turning points accounted for 83% of the total. The relationship between the occurrence of turning points and perceived training complexity was significant at: (1) pre-increase turning points in positive emotion trajectories (*p* = 0.003); and (2) pre-decrease turning points in negative emotion trajectories (*p* = 0.001), which was consistent with the first result.

## Discussion

We have organized our discussion around four issues: (1) The positive orientation of the students' emotional trajectories; (2) The relationship between contextual complexity and positively trended turning points in the students' emotional trajectories; (3) The concentration of turning points in the students' emotional trajectories around the middle of the training course; (4) The apparent scarcity of turning points in the students' emotional trajectories.

### The positive orientation of the students' emotional trajectories

Emotions play a key role in learning processes, since they enable us to make meaning of our experiences and adapt to our environment (Frijda, 1988; Lazarus, 1991; Solomon, 2007; Bradley, 2009). We therefore expected our participants' emotional experience to fluctuate over time as a result of their changing ways of making meaning of the new demands arising from the process-oriented experiential learning setting. The predominant emotional trajectory among our participants had a positive orientation, as evidenced by the two most frequent types of turning points: pre-increase turning points in positive emotion trajectories, and pre-decrease turning points in negative emotion trajectories. These turning points indicated that an initial response consisting of either increasingly intense negative emotions or decreasingly intense positive emotions was replaced by a pattern that consisted of decreasingly intense negative emotions or increasingly intense positive emotions.

On the one hand, the participants' prevalent initial experience of intense negative emotions and non-intense positive emotions in response to challenging demands is consistent with previous findings in higher education settings (see for example Apte, 2009; Dirkx, 2011). Dirkx (2008) argues that adults' emotional responses in learning settings are usually related to the content, the structure or the processes that they entail, so that an open structure can lead students to feel overwhelmed and to complain of a lack of direction. This is consistent with the likely experience of our participants.

Instead of the typical emphasis on learning protocols that would be expected in a didactic learning sequence, an experiential learning sequence places the emphasis on the students' exploration of their natural experience. This means that there is a predominance of open an exploratory exercises, and that no closed answers or procedures to follow are provided by the trainer. This is potentially challenging for many students, who are mostly used to content-based teaching practices (for related findings, see Nogueiras and Iborra, 2016), and can be a source of upsets. In addition to the new experiential learning sequence, something that can be upsetting for students is the content explored in the exploratory exercises. Newcomers may become upset at the beginning of the training by the mismatch between their expectations and the proposed learning sequence. Meanwhile, students who are used to the experiential learning sequence are expected to have a maximized set-up, since they already expect that they will not know from the beginning and know that they have to remain curious and open. For them, the possible upset would not be related to the experiential learning sequence, but instead to the possibility of dealing with complex experiential content.

On the other hand, the positive orientation of our participants' emotional trajectories over time is similar to that found by Arpiainen et al. (2013) in their research on students' emotions in an entrepreneurship learning program. In their thematic analysis of the students' in-depth interviews after the training, they found “waves of emotions” consisting of frequent negative emotions at the beginning of the program, and positive emotions toward the end. According to these authors, the students' negative emotional experience was a response to the new learning environment and to the challenging tasks they were set. Conversely, the positive shift in the students' emotional experience over time was considered to be related to their increased ability to cope with uncertainty during their learning process. This is something that we find also plausible in our study, as discussed in the paragraphs below.

In situations where our ways of meaning-making are destabilized, managing both the destabilization and the associated unpleasant emotions is necessary if learning is to occur (Taylor and Cranton, 2013). Our participants might have developed two different responses to the likely destabilization they experienced. One possibility is that participants were successful in their attempt to reduce the discrepancy between the demands of the training and their ways of meaning-making, so that they effectively managed their learning process. In this case, participants would have moved from a period of emotional discomfort to a period of emotional comfort (see Kunnen and Wassink, 2003). Another possibility is that participants were unable to manage the challenging demands of the training, so that the destabilization might have been counterproductive for learning, and undermined the participants' confidence in their way of creating meaning. In this case, the initial unpleasant emotional experience would have persisted or become more profound over time.

The predominantly positive orientation of our participants' emotional trajectories over time can be interpreted as evidence of their successful management of the challenging demands of the situation. This may in turn indicate a greater possibility for participants' cognitive accommodation. At this point, it is necessary to explicitly state what kind of cognitive accommodation we are referring to. To do this, it is possible to distinguish between two types of accommodation. On the one hand, an individual can change their cognitive structures when they understand something. In this case, the accommodation is focused on the content. On the other hand, the accommodation can be more focused on the process. In our case, we refer to accommodation that is related to the students' adaptation to the learning methodology, rather than to the content of learning itself. The former would involve a change in the way in which the participants adapted to the course, modifying their initial expectations and going beyond them. If this kind of accommodation took place, it would involve the participants changing their preference to a didactic learning sequence, and being more open to investigation and development in an experiential learning sequence. This would necessarily involve a higher degree of flexibility and tolerance of uncertainty.

In short, the two most common types of turning points found in emotional trajectories can be taken as an evidence of the students' positive set-down in response to the initial upset arising from the mismatch between their expectations and the training demands. After the students adapted to the new learning model, their emotional experience shifted from being predominantly unpleasant to being predominantly pleasant.

In view of the above, the study of emotional patterns over time in both Arpiainen et al.'s (2013) research and our own research supports the idea that a dynamic approach to the study of emotions enable to overcome the simplistic claim that positive emotions are good for learning and negative emotions are bad. Instead, and according to Sansone and Thoman's (2005) arguments, this study confirms that it is the dynamic patterns of positive and negative emotions over time, in connection with individuals' changing ways of managing contextual complexity, which can be considered positive or negative for learning. This last point is discussed further below.

### The relationship between contextual complexity and positively trended turning points in the students' emotional trajectories

The coincidence between positively trended turning points and particularly complex periods in the training is consistent with the idea that high levels of contextual complexity might act as a catalyst for individuals' new and more adapted behavioral patterns (Piaget, 1975/1985). If an experience is not challenging for individuals, they will not become involved in a meaning-making process aimed at creating a response that is adapted to their environment and the experience will therefore not be developmental. The positively trended turning points in the participants' emotional trajectories are an example of a developmental orientation, which leads us to assume that the complex experiential training triggered the participants' adaptation to the new contextual demands.

However, these assertions do not mean that contextual complexity always leads to experiences of upsets, and that those upsets lead to learning. Instead, if this happens it depends on how individuals manage complexity and the emotional upset associated with it. If the reasons for contextual complexity were emotional, all the participants' emotional trajectories would be similar, and this is not the case. Our hypothesis is therefore that there are other issues involved apart from complexity, such as the individuals' self-management baseline and the different paces and phases that individuals can follow over time when learning how to manage complexity, and which play an important role in learning. For example, some students may respond to contextual complexity by experiencing excitement and enjoyment, and not necessarily by experiencing unpleasant emotions. Thus, contextual complexity will not therefore always match upsets. Indeed, as people learn to manage complexity, emotional upsets may no longer be an issue. As mentioned above, students who expected complexity in the experience may have been less upset than students who did not expect complexity. In conclusion, we cannot generalize that complexity is upsetting for every individual, and neither can we state that it will inevitably lead to a successful adaptation to the context.

### The concentration of turning points in the students' emotional trajectories around the middle of the training course

In connection with the above, it is important to acknowledge that the contextual complexity identified at various points in the training may have led to different responses by the participants. If very high levels of complexity had been elicited at the beginning of the course, the participants' destabilization could have been too high to be managed successfully. However, the middle period of a training program is a more suitable time for participants to become destabilized. This is potentially because they are more prepared after a prior period of experience, in which when their ways of meaning-making might have been reorganized. From this vantage point, facing high complexity might have led to the emergence of a qualitatively different and more adapted emotional response, as indicated by positively trended turning points in the emotional trajectories. Interestingly, not only were more complex exercises likely to be proposed, but the participants' perception of complexity might also have varied over time. For example, at the beginning of the training the participants' complexity scores were not still very high probably because they were creating their own standards for the course, and not necessarily because the exercises proposed were less complex. This makes sense when we recall that a large percentage (63.33%) of the 30 turning points in emotional trajectories were located at m.p. 4 and at m.p. 6, and that furthermore, most of these turning points were positively oriented. The middle period of the training might therefore be considered the safest moment for the trainer to propose challenging input, both because students are likely to be more accustomed used to the learning sequence, and because they still have time to settle down in the context of the training course.

From the above, it can be concluded that contextual complexity alone is not a trigger for development and learning, but also that when this complexity is faced is also relevant in terms of the individuals' resources for managing it. When discussing identity development, Kroger (1993) interestingly points out that a certain readiness is needed if a conflict is to induce change in individuals. This is consistent with cognitive structural theory and research, which have provided evidence to suggest that individuals have to be at a certain stage for an optimal period of time before change is possible. Accordingly, in order to explain a qualitative change in participants' emotional response to the training demands, we have to take into account the prior process of reorganization and its subsequent impact. In dynamic systems, this is termed feedback delay (Van Geert, 1994; Kunnen, 2012).

### The apparent scarcity of turning points in the students' emotional trajectories

An issue arising from our findings that is also worth discussing is the apparent low number of turning points identified. We found 30 turning points in 272 emotional trajectories—87% of which were positively trended. However, we believe that this number is reasonable, as turning points in participants' emotional trajectories are taken as evidence of the reorganization of their prior ways of meaning-making. First, it must be acknowledged that in general, more stability than change would be expected, especially among adult students. Second, sometime is needed for this re-organization to take place. According to the literature on developmental turning points (Rönkä et al., 2002), as they are associated with changes in trajectories and internal reorganizations, sometime is needed to process these changes. Regardless of the timeframe of the nature of the trajectories studied (as highlighted by Lichtwarck-Aschoff et al., 2008, for example), which could amount to years (as in identity changes, e.g., Stevens, 2012), months (in the case of beliefs, attitudes, values, or commitment orientations, as in Kunnen et al., 2008) or even days (for specific patterns of behavior, creation of habits, and strategies, as in Siegler, 2006), turning points require some time to take place. We could therefore expect a low frequency of turning points.

Nevertheless, the number of exceptional points in the participants' emotional trajectories was quite high. There were 142 exceptional points in the 242 emotional trajectories analyzed, of which 70% were positively trended. Exceptional points provide also relevant information about the predominant shape of the emotional trajectories during the course. In this study, we have focused on the changes in the participants' emotional trajectories which were statistically significant, i.e., on turning points. However, as evidenced in the amount and quality of the exceptional points, the emotional orientation of most of the participants' trajectories is similar to the one signaled by the most common types of turning points: a positive orientation associated with points in time during the training which the participants perceived as particularly complex.

## Educational implications

Educational interventions that challenge individuals' ways of meaning-making are usually associated with emotional arousal (Kegan, 1994; Antonacopoulou and Gabriel, 2001; Cranton, 2002; Apte, 2009; McEwen et al., 2010). A detailed tracking of students' emotional trajectories over time in search of transition points marked by discontinuities—the turning points in our study—could therefore enable teachers to identify periods in training that might mobilize or inhibit students' adaptation and learning (see Hayes et al., 2007 for the same argument in the context of therapy). Similarly, a skilled teacher should take into account the periods when students are more open to change, in order to provide appropriate new input that destabilizes their current ways of meaning-making and facilitates the emergence of more adapted and complex ones (see Seligman, 2005; Thelen, 2005 for this argument in therapy contexts).

We therefore believe that learning contexts can be structured to support and guide students toward accommodation processes (for a similar argument referring to the role of therapy, see Kunnen and Wassink, 2003), so that they become deliberately developmental learning contexts (Kegan and Lahey, 2016). An endeavor for teachers in developmental learning contexts is to undertake a careful follow-up of how learners emotionally respond to the new demands during the training. Teachers must acknowledge that students may feel anxious, insecure or overwhelmed when immersed for the first time in a learning context which no longer provides them with the guidance they are used to. Awareness of the intra-individual variability of students, as evidenced in different emotional responses to a challenging learning context, is equally important in supporting individuals' learning. The same group may contain students who are able and willing to explore and enjoy new learning approaches, and students who are reluctant and afraid to do so. An issue to bear in mind is how open individuals are when responding to new inputs, i.e., how dominant and strong their patterns of meaning-making are, and how they interact with the new situation (Thelen, 2005). Kunnen and Bosma (2000) argue that individuals differ both in their preference for either accommodation or assimilation, and in their skills to apply these in a satisfactory manner, which leads to different learning and developmental trajectories. Accordingly, we predict that on the one hand, students with a greater preference for accommodation would be expected to present more turning points in their emotional trajectories, and particularly positively-trended turning points. On the other hand, students with greater preference for assimilation would be expected to present a more stable emotional experience, i.e., fewer turning points, or none.

## Limitations

This study has three main limitations: (1) the number and distribution of measurements; (2) the grouping of emotions into positive and negative; (3) the specificity of the sample.

As regards the number of measurements, the shortcoming involved in asking the students to assess their emotions at a few points in time is that some of their emotional fluctuations during the training was inevitably lost. This limitation is inherent in the study of any developmental process. Although the questionnaires were strategically distributed in order to increase the probability of capturing expected emotional upsets, the students could have experienced other upsets which they may have overcome by the time they completed the questionnaire. One possible way of recording these would entail asking the students at the end of every training session about the emotions they experienced most strongly, when they felt them and what they were related to.

It could also be argued that the distribution of measurements during the training course studies was not consistent. We agree that this is the case, but this was intentional since the goal was to obtain data from the students after the periods of the course that we expected would be most challenging for them. We therefore do not consider the issue of whether the follow-up questionnaires were filled in after a phase in which students had been sharing and reflecting on an experiential exercise, or the students had been doing an exploratory exercise, or the trainer had been giving instructions for the development of an exercise, to be a problem.

We acknowledge that the grouping of emotions into positive and negative emotions is an oversimplification of the students' emotional experience, which might lead to nuances in the patterns of discrete emotions being overlooked. However, considering the aim of this study, we found that valence sufficed for grasping the main emotional patterns.

Finally, it can be argued that the specificity of the sample may mean that the generalization of the results is questionable. It is important to note that our aim was not to generalize these results to other samples, but rather to examine a training course in which a process-oriented experiential learning model was implemented. In fact, the trainer of the course, John McWhirter, was the developer of the experiential learning model applied. Nevertheless, further research with different samples and in different learning settings is recommendable in order to explore possible differences in how students manage complexity.

## Directions for future research

This study can lead to the formulation of new research questions and hypotheses about how individuals manage contextual complexity. On the one hand, it is a first step in reviewing some of the traditional ideas about learning, such as the assumption that contextual complexity always leads to individuals' learning.

It also opens the door to further research on cognitive accommodation. This would require the inclusion of measures of students' learning performance. To do so, a learning setting in which performance could be followed over a much longer time span –several weeks at least- after potential turning points in students' emotional trajectories would be needed. Time is required before increases in individuals' performance become visible. In this study it was therefore not useful to include performance measures. From a systems perspective, transformations have to resettle, and often, there may be a short dip in performance shortly after the transformation, due to the system having to reorganize and getting used to new patterns.

The collection of qualitative data both throughout the learning experience and at the end of it is essential in an in-depth investigation on how different students make meaning of the challenging demands arising from a process oriented experiential learning model. The collection of time series data on variables such as the duration of the emotions experienced and the degree of challenge and support perceived during the training are also important for gaining greater insight into students' experience during a training course. This latter would provide more information than the variable of complexity used in this study.

Additionally, investigating traditional learning settings could show us whether the patterns in students' emotional trajectories differ from those found in a process-oriented experiential learning context. We anticipate that there would be no turning points in students' emotional trajectories, which would present a mostly flat shape. This would demonstrate that training based on didactic learning does not upset students and that it is therefore not a supportive context in terms of enhancing students' adaptation to complex contexts and providing greater opportunities for cognitive accommodation.

## Conclusion

Using a dynamic systems approach, this study examined the emotional trajectories of the participants in an experiential learning course in order to investigate how these individuals managed conflicting training demands. The most frequent types of turning points identified -pre-increases in positive emotion trajectories and pre-decreases in negative emotion trajectories- coincided with particularly complex periods in the training. The positive trend found in emotional trajectories is taken as evidence of the participants' adapted responses to the demands of the challenging learning approach.

These findings are consistent with the ideas that development and learning occur in response to conflicting demands that challenge individuals' ways of meaning-making, and that cognitive conflict is usually associated with the experience of negative emotions. However, our main contribution is providing empirical proof of these former claims, by establishing a dynamic systems procedure that identifies turning points in developmental trajectories. This procedure offers an alternative to standard research methods focusing on the product of development and which overlook the process, and confirms that dynamic systems are a very robust approach for achieving this. This procedure could be applied to a wide range of longitudinal experiences that are very common in educational and developmental settings.

Our findings also support the idea that contextual complexity alone does not lead to individuals' adaptation and learning. Instead, in order to explain this adaptation, we need to consider the way in which individuals manage contextual complexity. The quality and result of this management can be influenced by several factors, such as the particular individuals' self-management baseline, and the timing when the contextual complexity is faced in terms of the individuals' resources to manage it.

Finally, this study confirms that the process-oriented experiential learning model is particularly useful for supporting students in the process of managing contextual complexity. It provides students with a wide range of experiential variation and demands they engage in a different way of learning, in which exploration, curiosity and uncertainty are key factors. This is a potential opportunity for individuals to revise their previous ways of learning, which are usually rooted in didactic learning settings, and opens up the path to becoming more autonomous and creative learners.

## Ethics statement

This study was carried out in accordance with the recommendations of University of Alcalá Research Committee with written informed consent from all subjects. All subjects gave written informed consent in accordance with the Declaration of Helsinki. The protocol was approved by the University of Alcalá Research Committee.

## Author contributions

GN was responsible for research design, data collection, data analysis and writing of the manuscript. AI was responsible, together with GN, for research design and data collection. He also supported GN in the elaboration of the discussion and in the revision of the manuscript by providing feedback and comments. EK was responsible, together with GN, for data analysis. She also supported GN in the revision of the manuscript by providing feedback and comments.

## Funding

The first author of this paper was generously funded through a grant for the Training of University Teachers from the University of Alcalá (Spain).

### Conflict of interest statement

The authors declare that the research was conducted in the absence of any commercial or financial relationships that could be construed as a potential conflict of interest.

## Footnotes

^1^The general course of change over time in a variable is described as a “developmental trajectory” (Bosma and Kunnen, 2001).

^2^In the remainder of the article, and for the sake of brevity, we will use the label “experiential learning” to refer to a process-oriented experiential learning model as described by McWhirter (2002).

^3^Developmental Behavioural Modeling is a comprehensive field to systematically model modelling (McWhirter, 2002, 2011). It studies how human beings create our own models of the world through natural modelling skills, how effective our models are, and how we change them in order to adapt them to new circumstances in an optimal way (McWhirter, 1998).

^4^In a normal distribution, 1.65 standard deviations from the sample's mean represents around 90% of the population. We therefore assumed that a confidence level of 90% was reasonable for identifying exceptional points in the trajectories.

^5^The slope is a linear trend parameter that describes both the direction (increase or decrease) and the steepness (the strength of such decrease or increase) of the changes in the variable studied.

## References

[B1] Abbott A. (1997). On the concept of turning point. Comp. Soc. Res. 16, 85–106.

[B2] Antonacopoulou E. P. Gabriel Y. (2001). Emotion, learning and organizational change: towards an integration of psychoanalytic and other perspectives. J. Organ. Change Manage. 14, 435–451. 10.1108/EUM0000000005874

[B3] Apte J. (2009). Facilitating transformative learning: a framework for practice. Aust. J. Adult Learn. 49, 169–189.

[B4] Arpiainen R.-L. Lackéus M. Täks M. Tynjälä P. (2013). The sources and dynamics of emotions in entrepreneurship education learning process. Trames J. Human. Soc. Sci. 17, 331–346. 10.3176/tr.2013.4.02

[B5] Barrett L. F. (2009). Variety is the spice of life: a psychological construction approach to understanding variability in emotion. Cogn. Emot. 23, 1284–1306. 10.1080/02699930902985894 20221411 PMC2835153

[B6] Bosma H. A. Kunnen S. (2001). Determinants and mechanisms in ego identity development: a review and synthesis. Dev. Rev. 21, 39–66. 10.1006/drev.2000.0514

[B7] Bradley M. M. (2009). Natural selective attention: orienting and emotion. Psychophysiology 46, 1–11. 10.1111/j.1469-8986.2008.00702.x 18778317 PMC3645482

[B8] Camras L. A. Witherington D. C. (2005). Dynamical systems approaches to emotional development. Dev. Rev. 25, 328–350. 10.1016/j.dr.2005.10.002

[B9] Carver C. S. Scheier M. F. (1990). Origins and functions of positive and negative affect: a control–process view. Psychol. Rev. 97, 19–35. 10.1037/0033-295X.97.1.19

[B10] Cranton P. (2002). Teaching for transformation. New Direct. Adult Continuing Educ. 2002, 63–72. 10.1002/ace.50

[B11] Dirkx J. M. (2008). The meaning and role of emotions in adult learning. Emotion 2008, 7–18. 10.1002/ace.311

[B12] Dirkx J. M. (2011). Romancing tales from the dark side : crisis, emotion, and the construction of meaning in transformative learning, in 9th International Transformative Learning Conference - Athens 2011 - Book of Abstracts, 76–81.

[B13] Dirkx J. M. Mezirow J. Cranton P. (2006). Musings and reflections on the meaning, context, and process of transformative learning: a dialogue between John M. Dirkx and Jack Mezirow. J. Transform. Educ. 4, 123–139. 10.1177/1541344606287503

[B14] D'Mello S. Graesser A. (2011). The half-life of cognitive-affective states during complex learning. Cogn. Emot. 25, 1299–1308. 10.1080/02699931.2011.613668 21942577

[B15] D'Mello S. K. Craig S. D. Graesser A. C. (2009). Multimethod assessment of affective experience and expression during deep learning. Int. J. Learn. Technol. 4, 165–187. 10.1504/IJLT.2009.028805

[B16] D'Mello S. Lehman B. Pekrun R. Graesser A. (2014). Confusion can be beneficial for learning. Learn. Instruc. 29, 153–170. 10.1016/j.learninstruc.2012.05.003

[B17] Efron B. Tibshirani R. J. (1993). An Introduction to the Bootstrap. New York, NY: Chapman and Hall.

[B18] Eubanks-Carter C. Gorman B. S. Muran J. C. (2012). Quantitative naturalistic methods for detecting change points in psychotherapy research: an illustration with alliance ruptures. Psychother. Res. 22, 621–637. 10.1080/10503307.2012.693772 22708548

[B19] Fogel A. (2011). Theoretical and applied dynamic systems research in developmental science. Child Dev. Perspect. 5, 267–272. 10.1111/j.1750-8606.2011.00174.x

[B20] Fogel A. Nwokah E. Young Dedo J. Messinger D. Dickson L. K. Matusov E. (1992). Social process theory of emotion: a dynamic systems approach. Soc. Dev. 1, 122–142. 10.1111/j.1467-9507.1992.tb00116.x

[B21] Frijda N. H. (1986). The Emotions: Studies in Emotion and Social Interaction. Cambridge: Cambridge University Press.

[B22] Frijda N. H. (1988). The laws of emotion. Am. Psychol. 43, 349–358. 10.1037/0003-066X.43.5.349 3389582

[B23] Frijda N. H. (2009). Emotions, individual differences and time course: reflections. Cogn. Emot. 23, 1444–1461. 10.1080/02699930903093276

[B24] Good P. (1999). Resampling Methods: A Practical Guide to Data Analysis. Boston, MA: Birkhauser.

[B25] Granott N. Fischer K. W. Parziale J. (2002). Bridging to the unknown: a transition mechanism in learning and development, in Microdevelopment: Transition Processes in Development and Learning, eds Granott N. Parziale J. (New York, NY: Cambridge University Press), 3–33.

[B26] Hayes A. M. Laurenceau J. P. Feldman G. Strauss J. L. Cardaciotto L. (2007). Change is not always linear: the study of nonlinear and discontinuous patterns of change in psychotherapy. Clin. Psychol. Rev. 27, 715–723. 10.1016/j.cpr.2007.01.008 17316941 PMC3163164

[B27] Hayes A. M. Yasinski C. Ben Barnes J. Bockting C. L. H. (2015). Network destabilization and transition in depression: new methods for studying the dynamics of therapeutic change. Clin. Psychol. Rev. 41, 27–39. 10.1016/j.cpr.2015.06.007 26197726 PMC4696560

[B28] Hood G. M. (2010). PopTools Version 3.2.5. Available online at: http://www.poptools.org

[B29] Inzlicht M. Bartholow B. D. Hirsh J. B. (2015). Emotional foundations of cognitive control. Trends Cogn. Sci. 19, 126–132. 10.1016/j.tics.2015.01.004 25659515 PMC4348332

[B30] Kegan R. (1994). In Over Our Heads: The Mental Demands of Modern Life. Cambridge, MA: Harvard University Press.

[B31] Kegan R. Lahey L. L. (2016). An Everyone Culture: Becoming a Deliberately Developmental Organization. Boston, MA: Harvard Business Review Press.

[B32] Kloep M. Hendry L. B. Saunders D. (2009). A new perspective on human development. Conf. Int. J. Arts Sci. 1, 332–343.

[B33] Kolb A. Kolb D. (2009). Experiential learning theory: a dynamic, holistic approach to management learning, education and development, in The SAGE Handbook of Management, Learning, Education and Development, eds Armstrong S. J. Fukami C. (London: Sage Publications), 1–59.

[B34] Kolb D. A. (1984). Experiential Learning: Experience as the Source of Learning and Development. Englewood Cliffs, NJ: Prentice-Hall.

[B35] Kroger J. (1993). Discussions on Ego Identity. Hillsdale, NJ: Erlbaum.

[B36] Kunnen E. S. Sappa V. van Geert P. L. Bonica L. (2008). The shapes of commitment development in emerging adulthood. J. Adult Dev. 15, 113–131. 10.1007/s10804-008-9042-y

[B37] Kunnen S. (2006). Are conflicts the motor in identity change? Identity 6, 169–186. 10.1207/s1532706xid0602_3

[B38] Kunnen S. (2012). A Dynamic Systems Approach to Adolescent Development. London: Psychology Press.

[B39] Kunnen S. Bosma H. A. (2000). Development of meaning making: a dynamic systems approach. New Ideas Psychol. 18, 57–82. 10.1016/S0732-118X(99)00037-9

[B40] Kunnen S. Van Dijk M. Lichtwarck-Aschoff A. Visser M. Van Geert P. (2012). The search for variability and change, in The Search for Variability and Change in A Dynamic Systems Approach of Adolescent Development, ed Kunnen S. (London: Psychology Press), 53–71.

[B41] Kunnen S. Wassink M. E. K. (2003). An analysis of identity change in adulthood. Identity 3, 347–366. 10.1207/S1532706XID0304_03

[B42] Kuppens P. Oravecz Z. Tuerlinckx F. (2010). Feelings change: accounting for individual differences in the temporal dynamics of affect. J. Pers. Soc. Psychol. 99, 1042–1060. 10.1037/a0020962 20853980

[B43] Larsen R. J. Augustine A. A. Prizmic Z. (2009). A process approach to emotion and personality: using time as a facet of data. Cogn. Emot. 23, 1407–1426. 10.1080/02699930902851302

[B44] Lazarus R. S. (1991). Emotion and Adaptation. New York, NY: Oxford University Press.

[B45] Lepper M. R. Henderlong J. (2000). Turning “play” into “work”: 25 years of research on intrinsic versus extrinsic motivation, in Intrinsic and Extrinsic Motivation: The Search for Optimal Motivation and Performance, eds Sansone C. Harackiewicz J. M. (San Diego, CA: Academic Press), 257–307.

[B46] Lewis M. D. (2005). Bridging emotion theory and neurobiology through dynamic systems modeling. Behav. Brain Sci. 28, 169–194. 10.1017/S0140525X0500004X 16201458

[B47] Lichtwarck-Aschoff A. Kunnen S. Van Geert P. (2009). Here we go again: a dynamic systems perspective on emotional rigidity across parent-adolescent conflicts. Dev. Psychol. 45, 1364–1375. 10.1037/a0016713 19702398

[B48] Lichtwarck-Aschoff A. van Geert P. Bosma H. Kunnen S. (2008). Time and identity: a framework for research and theory formation. Dev. Rev. 28, 370–400. 10.1016/j.dr.2008.04.001

[B49] McEwen L. Strachan G. Lynch K. (2010). ‘Shock and Awe’ or ‘Reflection and Change’: stakeholder perceptions of transformative learning in higher education. Learn. Teach. High. Educ. 5, 34–55.

[B50] McWhirter J. (1998). Re-Modelling NLP Part One: Models and Modelling. Available online at: http://sensorysystems.co.uk/dbm-remodelled-nlp/part-one-models-and-modelling/

[B51] McWhirter J. (2000). Re-Modelling NLP. Part Six: Understanding Change. Available online at: http://sensorysystems.co.uk/dbm-remodelled-nlp/part-six-understanding-change-2/

[B52] McWhirter J. (2002). Re-Modelling NLP. Part Fourteen: Re-Modelling Modelling. Available online at: http://sensorysystems.co.uk/dbm-remodelled-nlp/re-modelling-nlp-part-fourteen-re-modelling-modelling/

[B53] McWhirter J. (2011). Behavioral remodeling, in Innovations in NLP: for Challenging Times, eds Hall L. M. Charvet S. R. Rose-Charvet S. (Carmarthen, UK: Crown House Publishing), 95–114.

[B54] Molenaar P. C. M. (2004). A manifesto on psychology as idiographic science: bringing the person back into scientific psychology, this time forever. Meas. Interdiscipl. Res. Perspect. 2, 201–218. 10.1207/s15366359mea0204_1

[B55] Noddings N. (2003). Happiness and Education. Cambridge: Cambridge University Press.

[B56] Nogueiras G. Herrero D. Iborra A. (2016). Teaching for epistemological change: self-direction through self-assessment, in Innovative Practices for Higher Education Assessment and Measurement, eds Cano E. Ion G. (Hershey, PA: IGI Global), 207–225.

[B57] Nogueiras G. Iborra A. (2016). Understanding and promoting self-direction in freshman and master's students: a qualitative approach. Behav. Dev. Bull. 10.1037/bdb0000024

[B58] Piaget J. (1952). The Origins of Intelligence in Children. New York, NY: International Universities Press.

[B59] Piaget J. (1975/1985). The Equilibrium of Cognitive Structures. Chicago, IL: University of Chicago Press.

[B60] Rönkä A. Oravala S. Pulkkinen L. (2002). “I met this wife of mine and things got onto a better track” Turning points in risk development. J. Adolesc. 25, 47–63. 10.1006/jado.2001.0448 12009749

[B61] Sansone C. Thoman D. B. (2005). Does what we feel affect what we learn? Some answers and new questions. Learn. Instruc. 15, 507–515. 10.1016/j.learninstruc.2005.07.015

[B62] Scherer K. R. (2009). The dynamic architecture of emotion: evidence for the component process model. Cogn. Emot., 23, 1307–1351. 10.1080/02699930902928969

[B63] Seligman S. (2005). Dynamic systems theories as a metaframework for psychoanalysis. Psychoanal. Dialogues 15, 285–319. 10.1080/10481881509348832

[B64] Siegler R. S. (2006). Microgenetic analyses of learning, in Handbook of Child Psychology, eds Damon W. Lerner R. M. (New York, NY: John Wiley and Sons), 464–510.

[B65] Solomon R. C. (2007). True to Our Feelings: What Our Emotions Are Really Telling Us. Oxford: Oxford University Press.

[B66] Stevens A. (2012). ‘I am the person now I was always meant to be’: identity reconstruction and narrative reframing in therapeutic community prisons. Criminol. Crim. Justice 12, 527–547. 10.1177/1748895811432958

[B67] Taylor E. W. Cranton P. (2013). A theory in progress? Issues in transformative learning theory. Euro. J. Res. Educ. Learn. Adults 4, 33–47. 10.3384/rela.2000-7426.rela5000

[B68] Thelen E. (1989). Self-organization in developmental processes: can systems approaches work?, in Systems and Development, eds Gunnar M. R. Thelen E. (New York, NY: Lawrence Erlbaum Associates), 77–107.

[B69] Thelen E. (2005). Dynamic systems theory and the complexity of change. Psychoanal. Dialogues 15, 255–283. 10.1080/10481881509348831

[B70] Thelen E. Smith L. B. (1994). A Dynamic Systems Approach to the Development of Cognition and Action. Cambridge, MA: MIT Press.

[B71] Thelen E. Ulrich B. D. (1991). Hidden skills: a dynamic systems analysis of treadmill stepping during the first year. Monogr. Soc. Res. Child Dev. 56, 1–98; discussion 99–103. 10.2307/1166099 1922136

[B72] Todman J. B. Dugard P. (2001). Single-Case and Small-N Experimental Designs: A Practical Guide to Randomization Tests. Mahwah, NJ: Lawrence Erlbaum Associates.

[B73] Vallacher R. R. Van Geert P. L. C. Nowak A. (2015). The intrinsic dynamics of psychological process. Curr. Dir. Psychol. Sci. 24, 58–64. 10.1177/0963721414551571

[B74] Van der Maas H. L. J. Molenaar P. C. (1992). Stagewise cognitive development: an application of catastrophe theory. Psychol. Rev. 99, 395–417. 10.1037/0033-295X.99.3.395 1502272

[B75] Van Dijk M. van Geert P. (2007). Wobbles, humps and sudden jumps : a case study of continuity, discontinuity and variability in early language development. Infant Child Dev. 16, 7–33. 10.1002/icd.506

[B76] Van Dijk M. van Geert P. (2011). Heuristic techniques for the analysis of variability as a dynamic aspect of change. Infancia Y Aprendizaje 34, 151–167. 10.1174/021037011795377557

[B77] Van Geert P. (1994). Dynamic Systems of Development: Change between Complexity and Chaos. New York, NY; London: Harvester Wheatsheaf.

[B78] Van Geert P. Steenbeek H. Kunnen S. (2012). Monte Carlo techniques. statistical simulation for developmental data, in A Dynamic Systems Approach to Adolescent Development, ed Kunnen S. (London: Psychology Press), 43–51.

[B79] Van Geert P. Van Dijk M. (2002). Focus on variability: new tools to study intra-individual variability in developmental data. Infant Behav. Dev. 25, 340–374. 10.1016/S0163-6383(02)00140-6

[B80] van Geert P. van Dijk M. (2015). The intrinsic dynamics of development, in Emerging Trends in the Social and Behavioral Sciences: An Interdisciplinary, Searchable, and Linkable Resource, eds Scott R. A. Kosslyn S. M. (Hoboken, NJ: Wiley), 1–16.

[B81] Weiss P. R. (2000). Emotion and learning. Train. Dev. 54, 44–48.

[B82] Witherington D. C. (2007). The dynamic systems approach as metatheory for developmental psychology. Hum. Dev. 50, 127–153. 10.1159/000100943

[B83] Yan Z. Fischer K. (2007). Pattern emergence and pattern transition in microdevelopmental variation: evidence of complex dynamics of developmental processes. J. Dev. Process. 2, 39–62.

