# Supplementary material for: Managing Contextual Complexity in an Experiential Learning Course: A Dynamic Systems Approach through the Identification of Turning Points in Students' Emotional Trajectories
Source: Front Psychol. 2017 May 3;8:667. doi: 10.3389/fpsyg.2017.00667 (PMC5414386; doi:10.3389/fpsyg.2017.00667)
Supplement: Supplementary file 1 [file DataSheet1.pdf]

Nogueiras, Kunnen, & Iborra (2017). Managing contextual complexity in an experiential learning course: a dynamic systems approach through the identification of turning points in students' emotional trajectories. Frontiers Psychology. http://journal.frontiersin.org/article/10.3389/fpsyg.2017.00667/abstract

Appendix 1 Overview of the timeframe of the training course. The follow-up questionnaires are boldfaced.

	Day 1	Day 2	Day 3	Day 4	Day 5
10.00 – 11.30	Course introduction<br>Trainer's *sets* students *up* and then presents instructions for exercise 1<br>Students do exercise 1<br>Trainer's *set-up* and instructions for exercise 2<br>Students do exercise 2<br>Students' sharing and reflection (*set-down*, consolidating learning)<br>Trainer's *set-up* and instructions for exercise 3<br>Students do Exercise 3<br>Trainer's *set-up* and instructions for exercise 4 (for the break)	Students' revision<br>Students' sharing and reflection (*set-down*, consolidating learning)<br>Trainer's *set-up* and instructions for exercise 1<br>Students do exercise 1<br>Trainer's *set-up* and instructions for exercise 2<br>Students do exercise 2<br>Trainer's *set-up* and instructions for exercise 3<br>Students do exercise 3	Students' revision<br>Trainer's *set-up* and instructions for exercise 1<br>**Questionnaire 5**<br>Students do exercise 1<br>Trainer's *set-up* and instructions for exercise 2 (for the break)	Students' revision<br>Students' sharing and reflection (*set-down*, consolidating learning)<br>**Questionnaire 7**<br>Trainer's *set-up* and instructions for exercises1 and 2<br>Students do exercises 1 and 2<br>Trainer's *set-up* and instructions for exercise 3<br>Students do exercise 3	Students' revision<br>Trainer's *set-up* and instructions for exercise 1<br>Students do exercise 1<br>Trainer's *set-up* and instructions for exercise 2<br>Students do exercise 2
			Coffee break		
12.00 – 14.00	Students' sharing and reflection (*set-down*, consolidating learning)<br>Trainer's *set-up* and instructions for exercise 5<br>Students do exercise 5<br>Trainer's *set-up* and instructions for exercise 6<br>Students do exercise 6<br>Students' sharing and reflection (*set-down*, consolidating learning)<br>**Questionnaire 1**	Trainer's *set-up* and instructions for exercise 4<br>Students do exercise 4<br>Students' sharing and reflection (*set-down*, consolidating learning)<br>Trainer's *set-up* and instructions for exercise 5<br>Students do exercise 5<br>Students' sharing and reflection (*set-down*, consolidating learning)<br>**Questionnaire 3**	Trainer's *set-up* and instructions for exercise 3<br>Students do exercise 3<br>Trainer's *set-up* and instructions for exercise 4<br>Students do exercise 4	Trainer's *set-up* and instructions for exercise 4<br>Students do exercise 4<br>Trainer's *set-up* and instructions for exercise 5<br>Students do exercise 5<br>Students' sharing and reflection (*set-down*, consolidating learning)	Trainer's *set-up* and instructions for exercise 3<br>Students do exercise 3<br>Trainer's *set-up* and instructions for exercise 4<br>Students do exercise 4<br>Students' sharing and reflection (*set-down*, consolidating learning)<br>Trainer's *set-up* and instructions for exercise 5<br>Students do exercise 5<br>Students' sharing and reflection (*set-down*, consolidating learning)<br>Trainer's *set-up* and instructions for individual exercise
			Lunch break		*End of the course*
16.00 – 18.00	Trainer's *set-up* and instructions for exercise 7<br>Students do exercise 7<br>Students' sharing and reflection (*set-down*, consolidating learning)<br>Trainer's *set-up* and instructions for exercise 8<br>Students do exercise 8<br>Students' sharing and reflection (*set-down*, consolidating learning)<br>Trainer's *set-up* and instructions for exercise 9 (for the break)	Trainer's *set-up* and instructions for exercise 6<br>Students do exercise 6<br>Students' sharing and reflection (*set-down*, consolidating learning)<br>Trainer's *set-up* and instructions for exercise 7<br>Students do exercise 7<br>**Questionnaire 4**	Trainer's *set-up* and instructions for exercise 5<br>Students do exercise 5<br>Trainer's *set-up* and instructions for exercise 6<br>Students do exercise 6<br>Students' sharing and reflection (*set-down*, consolidating learning)	Trainer's *set-up* and instructions for exercise 5.2<br>Students do exercise 5.2<br>Trainer's *set-up* and instructions for exercise 6<br>Students do exercise 6<br>Students' sharing and reflection (*set-down*, consolidating learning)	
			Coffee break		
18.30 – 20.00	Trainer's *set-up* and instructions for exercise 10<br>Students do exercise 10<br>**Questionnaire 2**	Students' sharing and reflection (*set-down*, consolidating learning)<br>Trainer's *set-up* and instructions for exercise 8<br>Students do exercise 8<br>Students' sharing and reflection (*set-down*, consolidating learning)	Trainer's *set-up* and instructions for exercise 7<br>Students do exercise 7<br>**Questionnaire 6**	Students' sharing and reflection (*set-down*, consolidating learning)<br>Students do exercise 7 in life<br>Trainer's *set-up* and instructions for exercise 8<br>Students do exercise 8<br>**Questionnaire 8**	
