# Supplementary material for: Managing Contextual Complexity in an Experiential Learning Course: A Dynamic Systems Approach through the Identification of Turning Points in Students' Emotional Trajectories
Source: Front Psychol. 2017 May 3;8:667. doi: 10.3389/fpsyg.2017.00667 (PMC5414386; doi:10.3389/fpsyg.2017.00667)
Supplement: Supplementary file 2 [file DataSheet2.pdf]

Nogueiras, Kunnen, & Iborra (2017). Managing contextual complexity in an experiential learning course: a dynamic systems approach through the identification of turning points in students' emotional trajectories. Frontiers Psychology. http://journal.frontiersin.org/article/10.3389/fpsyg.2017.00667/abstract

Appendix 2 Distribution of turning points (t.p.) across participants and across measurement points (m.p.). Pre-increase turning points in positive emotions are shaded light grey and pre-decrease turning points in negative emotions are shaded dark grey.

M.p. / Types t.p.	M.p. 2				M.p. 3				M.p. 4				M.p. 5				M.p. 6				M.p. 7					
	Pre-decrease t.p.		Pre-increase t.p.		Pre-decrease t.p.		Pre-increase t.p.		Pre-decrease t.p.		Pre-increase t.p.		Pre-decrease t.p.		Pre-increase t.p.		Pre-decrease t.p.		Pre-increase t.p.		Pre-decrease t.p.		Pre-increase t.p.			
Particip.	Pos. Emo.	Neg. Emo.	Pos. Emo.	Neg. Emo.	Pos. Emo.	Neg. Emo.	Pos. Emo.	Neg. Emo.	Pos. Emo.	Neg. Emo.	Pos. Emo.	Neg. Emo.	Pos. Emo.	Neg. Emo.	Pos. Emo.	Neg. Emo.	Pos. Emo.	Neg. Emo.	Pos. Emo.	Neg. Emo.	Pos. Emo.	Neg. Emo.	Pos. Emo.	Neg. Emo.	Total no. t.p.	Nº m.p. with t. p.
1																										
2																										
3		1						1																	2	2
4																	1								1	1
5										2	2														4	1
6						1																			1	1
7																										
8												2													2	1
9																		1							1	1
10		1																							1	1
11		3																							3	1
12											2														2	1
13																										
14						2				1									3						6	3
15																		2	3						5	1
16	1																								1	1
17														1											1	
Total no. t.p.	1	5				3		1		3	4	2		1			1	3	6						30	
No. participants with t.p.	1	3				2		1		2	2	1		1			1	2	2							
