# Supplementary material for: Managing Contextual Complexity in an Experiential Learning Course: A Dynamic Systems Approach through the Identification of Turning Points in Students' Emotional Trajectories
Source: Front Psychol. 2017 May 3;8:667. doi: 10.3389/fpsyg.2017.00667 (PMC5414386; doi:10.3389/fpsyg.2017.00667)

Nogueiras, Kunnen, & Iborra (2017). Managing contextual complexity in an experiential learning course: a dynamic systems approach through the identification of turning points in students' emotional trajectories. Frontiers Psychology. http://journal.frontiersin.org/article/10.3389/fpsyg.2017.00667/abstract

Appendix 3 Trajectories of complexity scores for each student over the 8 measurement points. Three additional trajectories are displayed: 1) highest complexity score, 2) average scores of complexity, and 3) lowest complexity score.

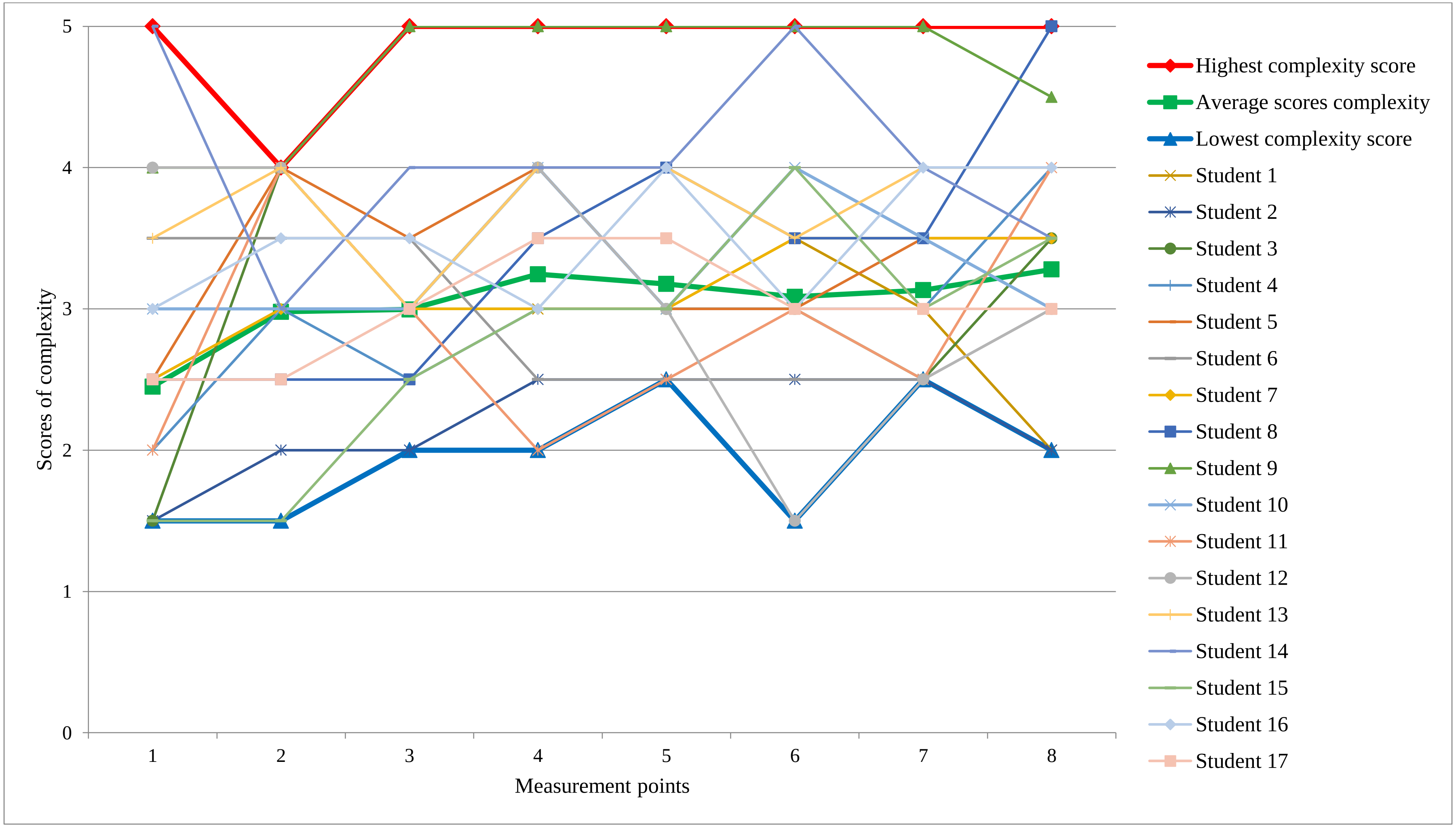

Supplement: Supplementary file 3 [file DataSheet3.pdf]
